# Special Issue on “Pleiotropic Benefits of Melatonin: From Basic Mechanisms to Disease”

**DOI:** 10.3390/ijms24065223

**Published:** 2023-03-09

**Authors:** Yaiza Potes, Beatriz Caballero

**Affiliations:** 1 Department of Morphology and Cell Biology, Faculty of Medicine, University of Oviedo, 33006 Oviedo, Asturias, Spain; 2 Instituto de Investigación Sanitaria del Principado de Asturias (ISPA), 33011 Oviedo, Asturias, Spain; 3 Instituto de Neurociencias del Principado de Asturias (INEUROPA), 33006 Oviedo, Asturias, Spain

Melatonin (N-acetyl-5-methoxytryptamine) is a multifunctional hormone that is naturally produced from tryptophan and released rhythmically throughout the night by the pineal gland to regulate sleep–wake cycles [1]. Melatonin is known as a messenger of darkness, given that its secretion increases after the onset of darkness, reaching peak levels in the middle of the night, and then gradually decreasing in the second half of the night [2]. Interestingly, the study of Martyniuk and collaborators (2022) also shows changes in the metabolic profile of melatonin-synthesis-related indoles that occur during post-embryonic development of the turkey pineal organ. Specifically, this study demonstrates that the concentration of melatonin increases until it reaches maximal levels at the age of 4 weeks in the turkey pineal organ and then remains at a rather constant level until the age of 45 weeks [3].

We should note that melatonin exerts other relevant cellular functions given its antioxidant and anti-inflammatory potential as well as its ability to regulate cell death processes [4]. Therefore, in addition to circadian and sleep modulation, melatonin plays several important roles in various biological processes, including ageing, stress response and immunity [2]. Thus, this Special Issue focuses on the pleiotropic benefits of melatonin, ranging from the basic mechanisms of its cell interaction to its beneficial effects under different disease conditions.

Melatonin mainly mediates its effects through its MT1 and MT2 membrane receptors, which belong to the superfamily of G protein-coupled receptors [5]. Interestingly, several other intracellular binding sites for melatonin have been described, including the enzyme NRH:quinone oxidoreductase 2 (NQO2), transcription factors such as retinoid orphan receptors (RORs), among which RORα is of particular importance, and intracellular proteins such as calretinin, tubulin and calmodulin [6]. In this regard, Argueta and collaborators (2022) provide new evidence regarding the direct interaction between melatonin and calmodulin and its effects on calmodulin-dependent kinase II (CaMKII) activity. Specifically, this relevant study has demonstrated that melatonin colocalizes in vivo with Ca^2+^-calmodulin in the hippocampus of mice injected with doses of melatonin that mediate antidepressant-like effects. Interestingly, these authors also show that calmodulin adopts different conformations during its interaction with melatonin, depending on the aqueous or lipid nature of the microenvironment, and with opposite effects on the CaMKII activity [7].

Yoo and Joo (2023) provide an update regarding other relevant intracellular targets of melatonin. In particular, CHOP (C/EBP Homologous Protein, also known as DNA damage-inducible transcript 3), GRP78/BiP (78-kDa Glucose-Regulated Protein/Binding immunoglobulin Protein), and PERK (Protein kinase R-like Endoplasmic Reticulum Kinase) have also been considered as potential intracellular targets for melatonin. These proteins are mediators of the endoplasmic reticulum’s stress responses, which are commonly activated in some neurological diseases, such as Alzheimer’s disease and Parkinson’s disease. Thus, melatonin might be protective for these neurodegenerative conditions by regulating endoplasmic reticulum stress via these specific cellular targets [8].

It is of paramount importance to note that melatonin levels and its receptors significantly and gradually decline in several organs over the life-span [9,10], which may favor both the progression of ageing, and the increased risk of suffering different pathologies. In this regard, there are many and varied studies that show that the exogenous administration of melatonin can be beneficial for dealing with different diseases and pathological and/or degenerative conditions [9,11,12,13]. Among the different published works, the revision study carried out by Wang and collaborators (2022) is noteworthy, in which the current evidence of the application of melatonin not only on periodontal treatment, but also on periodontitis-related systemic comorbidities is discussed. Periodontitis is not just a local inflammatory lesion limited to the oral cavity but is also closely linked to systemic comorbidities, including lung dysfunctions, cardiovascular disease, cancer, insulin resistance, Alzheimer’s disease, and adverse pregnancy outcomes, among other negative consequences. Due to the anti-infection, anti-inflammation and antioxidant properties of melatonin, as well as its bone-remodeling capacities, this indolamine provides relevant beneficial effects, not only on periodontal health, but also on general health [14].

The therapeutic potential of melatonin acquires special relevance in the fight against common metabolic disorders, such as obesity [12]. The study of Oliveira-Abreu and collaborators (2021) updates the data provided regarding the therapeutic efficacy of melatonin in the treatment of neural complications induced by one of the other most common metabolic disorders worldwide, diabetes mellitus. In particular, the anti-inflammatory and antioxidant properties of melatonin are intimately involved in its benefits on diabetic neuropathies and retinopathies [15].

The anticancer properties of melatonin have also been extensively studied in recent years. The study of Wand and collaborators (2022) provides a comprehensive review of the updated potential benefits and the limitations of melatonin as a preventive and therapeutic agent for cancer treatment in its initiation, promotion, and progression phases, either alone or in combination with other anticancer drugs [16]. Particularly, these authors suggest that certain measures must be taken, among which it is worth mentioning (a) the development of new drug forms or drug carrier systems to improve melatonin’s bioavailability; (b) the clarification of the criteria for controlling sample collection time and sample sources; (c) the improvement in dose-optimization processes; (d) the long-term safety of melatonin in cancer patients and; and (e) the better understanding of the molecular mechanisms and the clinical relevance to provide further evidence for the clinical use of melatonin as an anticancer drug [16].

The therapeutic applications of melatonin in clinical practice are incredibly varied, even including improvements in assisted reproductive technology (ART) outcomes [17]. In this context, the article of Tamura and collaborators (2022) shows how melatonin alters the granulose cells transcriptome by modifying gene expression associated with the inhibition of cell death, T-cell activity, and the activation of steroidogenesis and angiogenesis. As a consequence of this, melatonin could improve the oocyte quality and increase the blastocyst formation rate in patients whose embryo development rates in the previous ART cycle were less than 50%, ultimately leading to better outcomes of their infertility treatments [17].

The application of machine data learning technology in biomedicine is currently in full growth. For instance, we recently identified prognostic networks by applying this technology that are key for unraveling the biological mechanisms of sarcopenia [18]. Interestingly, Campos and collaborators (2023) applied machine data learning technology to identify those melatonin targets for a triad of psychosocial/sleep-circadian/cardiometabolic disorders. This novel study suggests the urgent need of obtaining major substantial evidence regarding the potential therapeutic use of melatonin on cardiovascular and metabolic disorders [19].

Recently, we have demonstrated that melatonin induces a recovery in the functionality of adult hippocampal neurogenesis in aged and neurodegenerative brains of mice with accelerated senescence [13]. In this respect, Sun and collaborators (2022) also showed that melatonin promotes antler growth in sika deer by accelerating MT1-mediated mesenchymal cell differentiation and inhibiting VEGF-induced degeneration of chondrocytes [20]. Given the impact of melatonin on cell differentiation processes, an important door is opened towards its potential on cell regeneration mechanisms that should be further investigated.

We have recently revised benefits of the neurogenic potential of melatonin for treating neurological and neuropsychiatric disorders under which processes related to adult brain neurogenesis are affected [21]. In particular, melatonin can modulate neurogenesis and produce an improvement in the brain functions under conditions of stress, anxiety, depression, dementia, in an ischemic brain, after a brain stroke or traumatic brain injury and under conditions of epilepsy, schizophrenia, amyotrophic lateral sclerosis and Down syndrome [21]. 

In summary, this Special Issue contains nine papers, including five original research articles and five systematic review articles. All of them provide important data related to the benefits of melatonin in several common pathological conditions, including diabetic neuropathy and retinopathy, cancer, neurodegenerative diseases, and fertility problems as well as under local and systemic inflammatory conditions. Melatonin plays a key role in different cellular signaling pathways, such as cell differentiation processes and gene expression, and it modulates different physiological mechanisms (e.g., oxidative stress and inflammation) with high efficacy and low toxicity. Therefore, is paramount to rigorously determine those basis mechanisms that melatonin may modulate in order to support the utility of melatonin in the therapy of these different pathological conditions in clinical practice.
